# The Impact of Single Nucleotide Polymorphisms on the Pharmacokinetics of Tacrolimus in Kidney Allograft Recipients of Northern-West, Iran

**DOI:** 10.34172/apb.2023.038

**Published:** 2022-01-02

**Authors:** Elaheh Jabbari Hagh, Ali Mousavi, Seyyedeh Mina Hejazian, Mehdi Haghi, Samaneh Esfahanian, Elham Ahmadian, Sepideh Zununi Vahed, Mohammadreza Ardalan

**Affiliations:** ^1^Kidney Research Center, Faculty of Medicine, Tabriz University of Medical Sciences, Tabriz, Iran.; ^2^Tuberculosis and Lung Diseases Research Center, Tabriz University of Medical Sciences, Tabriz, Iran.; ^3^Student Research Committee, Tabriz University of Medical Sciences, Tabriz, Iran.; ^4^Department of Animal Biology, Faculty of Natural Sciences, University of Tabriz, Tabriz, Iran.

**Keywords:** CYP 3A5 gene, Polymorphism, Tacrolimus, Kidney transplantation, PXR gene

## Abstract

***Purpose:*** Calcineurin inhibitors (CNIs) such as tacrolimus are a major immunosuppressive therapy after renal transplantation, which inhibit cytokine expression. The pharmacokinetics of such drugs is influenced by cytochrome P450 (CYP) enzymes, multi-drug resistance-1 (MDR-1), and C25385T pregnane X receptor (PXR). This study aimed to investigate the impact of single nucleotide polymorphisms (SNP) in these genes on the ratio of tacrolimus level per drug dosage (C/D ratio), acute graft rejection, and viral infections.

***Methods:*** Kidney transplantation recipients (n=65) under similar immunosuppressive treatment were included. Amplification refractory mutation systempolymerase chain reaction (ARMS-PCR) method was applied to amplify the loci containing the SNPs of interest.

***Results:*** Overall, 65 patients with a male/female ratio of 37/28 were included. The mean age was 38±1.75 years. The variant allele frequencies of CYP3A5*3, MDR-1 C3435T, and PXR C25385T were 95.38, 20.77, and 26.92%, respectively. No significant correlations were found between the studied SNPs and the tacrolimus C/D ratios. However, there was a significant difference in the C/D ratios at 2 and 8 weeks in homozygote CYP3A5 *3/*3 carriers (*P*=0.015). No significant association was found between the studied polymorphisms and viral infections and acute graft rejection (*P*>0.05).

***Conclusion:*** Homozygote CYP3A5 *3/*3 genotype could influence the tacrolimus metabolism rate (C/D ratio).

## Introduction

 Calcineurin inhibitors (CNI) are administered in 96% of allograft recipients and provided significant benefits in short-term graft survival.^1^ However, it is shown that the long-term survival of the graft is limited by the nephrotoxicity of these drugs.^2^ In a 10-year histopathologic follow-up of the transplanted kidney grafts, the use of CNIs was associated with decreased early subclinical rejection while progressive arteriolar hyalinosis, glomerulosclerosis, and tubulointerstitial damage were observed.^3^ Although tacrolimus, in contrast to cyclosporine, causes less pathological changes in the transplanted allografts within the first year, its pathological insults were similar to those of cyclosporine’s after a long time.^4^ Since tacrolimus has therapeutic benefit with detrimental effects on graft survival, understanding its pharmacokinetics and interactions is important.^5^

 Tacrolimus is metabolized by cytochrome P450 (CYP) 3A4 and 3A5 enzymes and variations in their coding genes were shown to be associated with altered drug clearance.^6^ Patients with high clearance of tacrolimus at first 90 days after transplantation are at an increased risk of acute graft rejection.^7^ CYP 3A4*22 T carriers require fewer doses of tacrolimus.^8^ The CYP 3A5*1 allele, which encodes the functional enzyme, causes a two-fold decrease in dose-normalized plasma levels of tacrolimus.^9^ This allele is more frequent among the patients with African-Americans ethnicity and associated with a 50% increase in total dosage requirements of tacrolimus to reach the first therapeutic trough level.^10^ In a study, among 337 renal allograft recipients, the presence of the CYP 3A4*22 allele and expression of CYP 3A5 were predictors of 20% and 160% clearance fluctuation, respectively.^11^ Thus, a personalized medicinal approach is necessary to determine the tacrolimus dosing to maintain therapeutic range in renal allograft recipients.

 The A6986G (rs776746) single nucleotide polymorphism (SNP) in the *CYP 3A*5 gene has been a focus of researchers during recent years. This SNP is located on the 3^rd^ intron of the gene and impacts the CYP 3A5 expression.^12^ However, there are conflicting findings among the studies. Additionally, two other SNPs including the C3435T of multi-drug resistance-1 (MDR-1) and C25385T pregnane X receptor (PXR, also known as nuclear receptor 112) genes were also investigated for their impact on the pharmacokinetics of tacrolimus. The obtained results are widely different across populations with different genetic backgrounds.

 In the clinics, kidney recipients differently respond to a given pharmacological therapy, some of whom cannot reach the designated concentration(s) with recommended starting doses. The over-dosing of the drug increases the risks of nephrotoxicity, infection, and other severe drug-specific side effects, while its under-dosing may lead to the acute rejection. Hence, the CNIs management is a challenging issue in transplant patients. Considering that tacrolimus is widely used in transplant immunosuppression and there are inter-individual differences in respond to the drug, understanding the patients’ pharmacogenetics and personalized administration of the drug could be helpful in different populations. Therefore, in this study, the influence of the *CYP*3A5, *MDR*-1, and *PXR* genes SNPs on the pharmacokinetics of tacrolimus and the ratio of concentration per dose of tacrolimus (C/D ratio) were evaluated in a group of kidney recipients in Northwest of Iran.

## Methods

###  Patients

 In this cross-sectional study, renal allograft recipients receiving tacrolimus were included. During the study period, renal transplant recipients with an age range of 20-60 years under triple therapy with tacrolimus (Prograf^®^ manufactured by Astellas Company, in 2 divided doses), mycophenolate mofetil (MMF), and prednisolone were included. Patients with ongoing acute allograft rejection, BK virus nephropathy, hepatitis, and cytomegalovirus (CMV) infections were excluded from the study.

###  Laboratory examinations

 A baseline laboratory examination including plasma creatinine, blood urea nitrogen, lipid profile, total bilirubin, and 25-hydroxy vitamin D levels were measured. The measurement of plasma levels of tacrolimus trough level was performed exactly 12 hours after the last dose of the drug and immediately before taking the next dose. Tacrolimus metabolism rate (C/D ratio) was calculated by dividing the blood concentration of the drug (C, ng/mL) to the daily dose of Tacrolimus (D, mg) at 2, 4 and 8 weeks after transplantation.

 The genomic DNA was extracted from blood with the magnetic nanoparticle-based method using ZiAViZ^®^ DNA extraction kit (Itan). Amplification refractory mutation systempolymerase chain reaction (ARMS-PCR) method was performed using primers (Table 1) designed based on previous studies.^13,14^ The commercial master mix kit (Sinaclon-Iran, #CatNo. MM2062) was used for the PCR procedure (Mastercycler Eppendorf, Germany).

**Table 1:** The sequencing of the primers used in PCR

**Primers**	**Type**	**Sequence (5’→3’)**
CYP 3A5 (A6986G)	Forward	CACTTGATGATTTACCTGCCTTC
	Wild-type reverse	GGTCCAAACAGGGAAGAGATAA
	Mutant reverse	GGTCCAAACAGGGAAGAGATAC
MDR1 (C3435T)	Forward	ACTATAGGCCAGAGAGGCTGC
	Wild-type reverse	GTGGTGTCACAGGAAGAGCTT
	Mutant reverse	GTGGTGTCACAGGAAGAGCTC
NR1I2 (C25385T)	Forward	ACCACGATTGAGCAAACAGGTA
	Wild-type reverse	TGGTCATTTTTTGGCAATCCCAGGTTC
	Mutant reverse	TGGTCATTTTTTGGCAATCCCAGGTTT

###  Statistical analysis

 The Hardy-Weinberg equilibrium was checked for each SNP. IBM SPSS Statistics version 24.0 was used for statistical analysis. Descriptive statistics demonstrates the frequency, percentage, mean, and standard deviation (SD). A logistic regression model with a confidence interval (CI) of 95% was applied for the evaluation of the correlations among the variables. ANOVA or Kruskal-Wallis tests were applied to compare the differences between tacrolimus C/D ratio at different time points (2, 4, and 8 weeks). The chi-square test was applied to check the correlation between the viral infections and graft rejection *vs.* SNP genotypes. A *P* value of < 0.05 was considered significant.

## Results and Discussion

 In total, 65 renal recipients were included in the study with a male/female ratio of 37:28. The average age was 38 ± 14.17 years old. The demographic data of the patients are presented in Table 2. It is reported that in the CYP3A5 nonexpressers, genotyping of NR1I2/ MDR1/ CYP3A4 polymorphisms can be useful for controlling tacrolimus dosing.^15^ In the present study, the variant allele frequencies of CYP3A5*3, MDR-1 C3435T, and PXR C25385T were 95.38, 20.77, and 26.92%, respectively.

**Table 2:** The baseline demographic and laboratory data

**Variables**	**Values**
Gender (male/female)	37/28
Age (y)	38 ± 14.17
Body mass index (kg/m^2^)	24.23 ± 5.14
Height (cm)	162.47 ± 12.2
Weight (kg)	63.58 ± 14.83
Blood urea nitrogen (mg/dL)	56.38 ± 28.71
Creatinine (mg/dL)	1.11 (0.54-9.2)
Triglycerides (mg/dL)	143 (29-592)
Total cholesterol (mg/dL)	171.52 ± 49.39
High-density lipoprotein (mg/dL)	43.09 ± 7.38
Low-density lipoprotein (mg/dL)	92.5 (0-180)
25-Hydroxy vitamin D3 (ng/ml)	17.79 ± 13.22

###  Allele frequency and genotype distribution of the CYP3A5*3

Most of the kidney recipients (90.8%, n = 59) were homozygotes for the CYP3A5*3 allele (CYP3A5 *3/*3, non-expressers CYP3A5), while 9.2% of the participants (n = 6) were heterozygous for the CYP3A5*3 (CYP3A5 *1/*3 genotype, functional CYP3A5 expressers). Across different populations, the frequency of CYP3A5*3 SNP differs widely. In our kidney recipient population, the frequency of the CYP3A5*3 allele was 95%. The result of a meta‐analysis has stated that the allele frequency of CYP3A5*3 is higher in European (94%), admixed American (80%), East Asian (71%), and South Asian (67%) subjects, while it is low (33%) in African subjects.^16^ Another meta-analysis (2015) concluded that the CYP3A5 A6986G SNP could affect the pharmacokinetics of tacrolimus.^17^ In the carriers of the wild-type allele (CYP3A5*1, expresser genotype, fast metabolizers), the expression of CYP3A5 leads to an elevated metabolism and clearance of tacrolimus and a lower C/D ratio compared to the other genotypes (CYP3A5*1/*3 and CYP3A5*3/*3 carriers). South African kidney transplant recipients in comparison with global transplant populations have a high rate of CYP3A5 expression that significantly affects the pharmacokinetics of tacrolimus.^18^ However, some studies did not find a statistically significant correlation between the A6986G SNP and higher clearance of tacrolimus^19^ being in agreement with our study.

###  Allele frequency and genotype distribution of the MDR-1 C3435T

 The frequency of the C and T alleles in *MDR*-1 C3435T gene was 79.23 and 20.77%, respectively. The genotypes distribution and allele frequency of the studied polymorphism are presented in Table 3. The MDR-1 affects the pharmacodynamics of tacrolimus by active transporting of a wide variety of drugs to the outsides of the cells.^20^ Similar to the CYP3A5, wild-type carriers of at least one MDR-1 C allele are expected to express more P-gp. The MDR1 C3435T SNP is a synonymous mutation in which the ATC codon changes to ATT (Ile1145Ile), causing alteration in P-gp activity.^21-24^ A higher activity of the MDR-1 limits the enteral absorption of tacrolimus. Similar to the results of Loh et al,^25^ 67.7% of our recipients were homozygote C allele carriers in contrast to other reports.^26^ In addition, study by Tada et al^27^ revealed that the MDR1 C3435T polymorphism did not affect tacrolimus pharmacokinetics of renal transplanted patients. Among the Asian population, SNPs in the *MDR*-1 and *CYP*3A5 genes were shown to influence the plasma levels of tacrolimus but not cyclosporine. Moreover, it has been proposed that diltiazem, a non-dihydropyridine calcium channel blocker, may help achieve the optimum levels of tacrolimus by blocking the MDR-1 and CYP 3A4 proteins.^25^

**Table 3:** Allele and genotype frequencies of the studied polymorphisms in kidney recipients

**Polymorphisms**	**Genotype**	**Allelic status**	**Genotype frequencies, n (%)**	Allelic frequencies, n (%)*	
CYP 3A5 A6986G	*1/*1 (AA)	A	0 (0%)	**A**	**G**
	*1/*3 (AG)	A	6 (9.2%)	4.62%	95.38%
	*3/*3 (GG)	G	59 (90.8%)		
MDR-1 C3435T SNP	CC	C	44 (67.7%)	**C**	**T**
	CT	C	15 (23.1%)	79.23%	20.77%
	TT	T	6 (9.2%)		
PXR C25385T SNP	CC	C	40 (61.5%)	**C**	**T**
	CT	C	15 (23.1%)	73.08%	26.92%
	TT	T	10 (15.4%)		

PXR: pregnane X receptor. * Results were obtained based on https://wpcalc.com/en/equilibrium-hardy-weinberg/.

###  Allele frequency and genotype distribution of the PXR C25385T SNP

 The frequency of the C allele in the *PXR* genes was 73.08%. The genotypes distribution and allele frequency of this SNP are presented in Table 3. The *PXR* gene, encoding a nuclear receptor, is involved in the regulation of CYP3A enzymes, MDR-1, and other several proteins and therefore, plays a crucial role in the pharmacokinetics of tacrolimus.^28^ However, there are contradictions about its effect on the pharmacokinetics of tacrolimus.^29^ This study revealed that the frequency of the PXR C25385T allele was 26.92%.

###  The correlation between clinical variables and gene polymorphisms

 The median (min-max) C/D ratio (ng/mL/mg) for the kidney recipients at 2, 4, and 8 weeks were 1.2 (0.13-5), 1.68 (0.32-10.67), and 2 (0.33-13), respectively. There was a significant difference in C/D ratios at 2 and 8 weeks (P = 0.015). In addition, the C/D ratios of patients with different genotypes in the studied genes are given in Table 4. The mean of C/D ratios in the non-expresser group compared to the expresser group were 1.56 ± 1.1 *vs*. 0.83 ± 0.16 ng/mL/mg at 2 weeks, 2.01 ± 1.0 *vs*. 1.75 ± 0.91 ng/mL/mg at 4 weeks, and 2 *vs*. 1.2 at 8 weeks after renal transplantation, respectively. There was a significant difference when C/D ratios of homozygote CYP3A5 *3/*3 carriers were compared between 2 and 8 weeks (*P* = 0.012).

**Table 4:** The C/D ratios in 2^nd^, 4^th^ and 8^th^ weeks of tacrolimus administration in relation to SNP genotypes

**SNPs**	**Genotype**	**Week 2**			**Week 4**			**Week 8**			***P*** value*
		**Concentration**	**Daily dose**	**C/D ratio**	**Concentration**	**Daily dose**	**C/D ratio**	**Concentration**	**Daily dose**	**C/D ratio**	
CYP 3A5 A6986G	*1/*1	**-**	**-**	**-**	**-**	**-**	**-**	**-**	**-**	**-**	0.217^a^
	*1/*3	5.66±0.57	7±1.73	0.83±0.16	10±3.6	6±1	1.75±0.91	9±4.35	5.33±0.57	1.2 (1.2-2.8)	**0.01** ^b^
	*3/*3	7.96±4.07	5.71±2.2	1.56±1.1	9.95±4.68	4.97±2.08	2.01±1.0	8.68±3.22	4.55±2.17	2 (0.33-13)	0.99^c^
MDR-1 C3435T	CC	7.71±3.68	5.66±2.38	1.57±1.03	9.07±4.88	5.03±2.24	1.66 (0.5-10.67)	8.73±3.26	4.79±2.39	2.23±1.28	0.88^a^
	CT	9 (2-20)	5.77±1.64	1.25 (0.29-5)	8.11±4.45	4.88±1.83	1.69±0.6	8.77±3.19	3.83±1.62	2 (1-13)	0.13^b^
	TT	4.8±1.09	6.6±2.07	0.8±0.4	6.32±2.83	5.4±1.34	1.28 (0.33-2)	8.44±4.03	5±1.22	1.72±0.83	0.88^c^
PXR C25385T	CC	6.46±2.79	5.48±1.76	1.25 (0.29-4.5)	8.6±3.85	5.01±1.62	1.66 (0.33-10.6)	8.67±3.31	4.59±1.42	2 (0.6-4.6)	1.01^a^
	CT	9.8±5.67	6.5±3.13	1.35 (0.4-5)	6.2 (3-22)	4 (2-12)	1.42 (0.5-4.86)	7.99±3.45	3.25 (2-12)	2 (0.33-4)	0.13^b^
	TT	9.25±3.68	6.25±2.06	1.48±0.34	6.7±1.24	4.25±0.5	1.61±0.42	10.72±1.74	3.12±1.43	2.88 (2.2-13)	0.13^c^
**Total**		6 (1-23)	5.66±2.07	1.2 (0.13-5)	7.2 (1.9-32)	4.95±1.95	1.68 (0.32-10.67)	8.82±3.49	4.42±2.04	2 (0.33-13)	0.19^a^
											**0.01**^b^
											0.93^c^

Daily dose: mg/kg/d; Plasma concentration of Tacrolimus: ng/mL; C/D ratio (ng/mL)/(mg/kg/d). Data with normal distribution were shown as mean ± SD and while data with non-normal distribution were shown as median (min-max). ^a^ C/D ratio of at week 2 compared to week 4 among homozygote SNP careers. ^b^ C/D ratio at week 2 compared to week 8 among homozygote SNP careers. ^c^ C/D ratio at week 4 compared to week 8 among homozygote SNP careers. * Results were compared by one-way ANOVA followed by Tukey test or Kruskal-Wallis test. Statistically significant *P* values are indicated in bold.

 Six patients experienced acute graft rejection, none of the SNPs seemed to have a significant impact on rejection rates (*P*> 0.05). In addition, 10 patients experienced viral infections (CMV, BK virus, or both), however, none of the SNP genotypes had a significant predisposing influence on them (*P*> 0.05).

###  Pharmacogenetics and tacrolimus metabolism

 Based on the studied genotypes and C/D ratios after 2, 4, and 8 weeks, renal transplant recipients were categorized into three groups (Table 5) based on the rate of tacrolimus metabolism. The groups were included C/D ratio < 1.05 (ng/mL/mg)/ (mg/kg/d) as fast metabolizers, 1.05 < C/D ratio < 1.55 (ng/mL/mg)/ (mg/kg/d) as intermediate metabolizers, and C/D ratio > 1.55 (ng/mL/mg)/ (mg/kg/d) as slow metabolizers, Thölking et al.^30^

**Table 5:** Category of patients based on C/D ratio of Tacrolimus

**Polymorphisms**	**Genotype**	**Metabolizers, n (mean±SD)**		
		**Fast metabolizers**	**Intermediate metabolizers **	**Slow metabolizers **
CYP 3A5 A6986G SNP	*1/*1	-	-	-
	*1/*3	1 (1 ± 0.0)	1 (1.06 ± 0.0)	4 (2.29 ± 0.76)
	*3/*3	17 (0.78 ± 0.18)	7 (1.3 ± 0.12)	30 (2.78 ± 1.28)
MDR-1 C3435T SNP	CC	13 (0.76 ± 0.2)	7 (1.28 ± 0.16)	23 (2.62 ± 0.99)
	CT	2 (0.86 ± 0.12)	2 (1.33 ± 0.007)	9 (3.21 ± 1.74)
	TT	3 (0.91 ± 0.11)	1 (1.33)	2 (1.7 ± 0.16)
PXR C25385T SNP	CC	12 (0.82 ± 0.18)	7 (1.26 ± 0.11)	20 (2.69 ± 1.4)
	CT	4 (0.69 ± 0.2)	2 (1.44 ± 0.08)	8 (2.81 ± 0.61)
	TT	2 (0.86 ± 0.19)	1 (1.08)	6 (2.74 ± 1.42)
**Total**		19 (0.83 ± 0.22)	8 (1.24 ± 0.12)	34 (2.72 ± 1.23)

Fast metabolizers: C/D ratio < 1.05, Intermediate metabolizers: 1.05 < C/D ratio < 1.55), slow metabolizer: C/D ratio > 1.55.

 It is reported that the expresser genotype (with a C/D ratio < 1.05) is correlated with a higher risk of chronic nephrotoxicity and acute rejection and a lower estimated glomerular filtration rate (eGFR) compared to slow and intermediate tacrolimus metabolizers.^17,31,32^ In the present study, about half of the kidney recipients with CYP3A5*3/*3 genotype were slow-metabolizers, confirming the impact of other genes in the pharmacokinetics of tacrolimus.

 It is shown that the 3435 C/C genotype of *MDR*-1 gene is associated with 40% lower C/D ratios.^29^ Since drug concentrations accomplished with a standard dose will be lower for carriers of the wild-type C allele, C/D ratios is expected to be lower. Dissimilar to this expectation, our patients with the C allele had higher C/D ratios at week 2; 1.57 ± 1.03 for homozygotes and 1.25 (min-max 0.29-5) for heterozygotes versus 0.8 ± 0.4 T allele. The SNPs in *CYP*3As and *MDR*-1 genes are attracting attention in clinical practice.^33^ Our results showed that the frequency of the T allele was 20.77% among renal transplanted, 3 of the carriers were fast metabolizers with a 0.91 ± 0.11 C/D ratio and 2 patients were slow metabolizers with a 1.7 ± 0.16 C/D ratio. However, this polymorphism did no significant effect on the metabolism of tacrolimus.

 Kurzawsk et al demonstrated that minor T allele carriers (CT or TT genotype) of the PXR C25385T require significantly low tacrolimus dose administration particularly in the first 6 months after renal transplantation compared to CC genotypes.^34^ In the present study, 2 of fast metabolizers with PXR SNP TT genotype had a 0.86 ± 0.19 C/D ratio and 6 patients were slow metabolizers with a 2.74 ± 1.42 C/D ratio. There were no significant differences between the studied polymorphism and fast/ intermediate/slow metabolizers (Table 5).

 The nephrotoxicity of CNIs has led to the assumption that perhaps lowering the dosage of CNIs or utilizing a less toxic agent (e.g. belatacept, sirolimus) would be a safer option.^2,35^ On the contrary, the triple regimen of tacrolimus, MMF, and a corticosteroid is ubiquitously used at present.^36^ It is shown that lower plasma levels of tacrolimus with a higher dosage of MMF are associated with better outcomes.^37^ Scholten et al developed a flexible method for accurate dosing of tacrolimus regardless of the genetic variables which is an ongoing field of research.^38^ There are somewhat controversial findings from other studies. We believe that the differences among the methodologies, tacrolimus release (immediate or extended), and perhaps ethnic differences may lead to such results.

## Conclusion

 In this study, the frequency of the CYP3A5*3 allele was 95%. There was a significant difference in the C/D ratios at 2 and 8 weeks among homozygote CYP3A5 *3/*3 carriers, suggested that choosing the initial dosage according to the CYP3A5 genotype may result in a better outcome. No significant correlations were found between the *MDR*-1, and *PXR* gene SNPs and the tacrolimus C/D ratios. These result will be a step toward personalized medicine and may prolong the graft survival in renal allograft recipients. Pre-transplant genetic study of recipient could prevents low drug level rejection or high level nephrotoxicity to occur.

## Acknowledgments

 The authors greatly appreciate the Kidney Research Center, Tabriz University of Medical Sciences, Tabriz, Iran for scientific and financial support (Grant number: 59763). This paper was extracted from the residential thesis of Elaheh Jabbari-Hagh, MD. Authors also thanks Transplantation Ward of Imam Reza Hospital of Tabriz University of Medical Science, Tabriz, Iran.

## Competing Interests

 Authors declare no conflicts of interest regarding this manuscript.

## Ethical Approval

 The Helsinki Declaration of ethics in medical research was honored in this study. The informed written consent was obtained from all patients. The study was approved by the ethics committee of Tabriz University of Medical Sciences, Tabriz, Iran and is registered with the following ethical code: IR.TBZMED.REC.1397.128.
